# Prenatal Exposure to Tobacco and Childhood Cognition and Behavior: Effect Modification by Maternal Folate Intake and Breastfeeding Duration

**DOI:** 10.1007/s10578-023-01524-x

**Published:** 2023-04-08

**Authors:** Adrienne T. Hoyt, Anna V. Wilkinson, Peter H. Langlois, Carol A. Galeener, Nalini Ranjit, Dana M. Dabelea, Brianna F. Moore

**Affiliations:** 1 Department of Health Promotion and Behavioral Sciences, UTHealth School of Public Health – Austin Regional Campus, Austin, TX USA; 2 Department of Epidemiology, Human Genetics, and Environmental Sciences, UTHealth School of Public Health – Austin Regional Campus, Austin, TX USA; 3 Department of Management, Policy and Community Health, Fleming Center Health for Care Management Houston, UTHealth School of Public Health– Houston Regional Campus, Houston, TX USA; 4 Lifecourse Epidemiology of Adiposity and Diabetes (LEAD) Center, Colorado School of Public Health, Aurora, CO USA; 5 Department of Pediatrics, University of Colorado School of Medicine, Aurora, CO USA; 6 Department of Epidemiology, Colorado School of Public Health, Aurora, CO USA; 7 University of Colorado Anschutz Medical Campus, 1890 N Revere Ct, Aurora, CO 80045 USA

**Keywords:** Tobacco, Folate, Breastfeeding, Interaction, Neurodevelopment, Fetal origins

## Abstract

In this exploratory analysis, we assessed whether nutrition modified the association between prenatal exposure to tobacco and childhood cognition/behavior among 366 Colorado-based mothers and their offspring (born ≥ 37 weeks with birthweights ≥ 2500 g). Interaction by folate (</≥ 1074 µg/day) and breastfeeding (</≥ 5 months) was assessed by including a product term with cotinine (</≥ limit of detection [LOD]) in regression models for NIH Toolbox and Child Behavior Checklist T-scores. Main effects were observed between cotinine ≥ LOD and inhibitory control (− 3.2; 95% CI: − 6.8, 0.3), folate < 1074 µg/day and anxious/depressed symptoms (1.1; 95% CI: 0.1, 2.1), and breastfeeding < 5 months and receptive language (− 4.3; 95% CI: − 8.5, − 0.02), though these findings would not survive Bonferroni correction. Breastfeeding modified the tobacco-behavior associations. Sleep (3.8; 95% CI: 0.5, 7.1; interaction p-value = 0.02), depressive (4.6; 95% CI: 1.0, 8.2; interaction p-value = 0.01) and total problems (5.8; 95% CI: − 0.7, 12.4; interaction p-value = 0.09) were observed among tobacco-exposed offspring who breastfed > 5 months, but not for shorter durations. Our findings support the need for smoking cessation campaigns throughout pregnancy and throughout the postpartum period breastfeeding to reduce neurobehavioral risks in the offspring.

## Introduction

Prenatal exposure to tobacco is associated with systematic growth restriction of the offspring, including low birth weight and a smaller head circumference [1]. Epidemiologic studies, including results from our own cohort, suggest that these growth-restricted offspring go on to experience cognitive and behavioral problems in early childhood [2–5]. These findings are supported by animal models, which show that prenatal exposure to nicotine is associated with hyperactivity, decreased attention, and mild learning deficits in the offspring [6–8].

The neurocognitive burden of prenatal exposure to tobacco is concerning, as many tobacco exposures during pregnancy are involuntary. Estimates from 2014 suggest that nearly one in four non-smoking adults of childbearing age experience secondhand exposure to tobacco [9]. Thus, there is a need to identify individual-level, nutritional factors that may mitigate the adverse programming caused by prenatal exposures to tobacco. Identification of such mitigating factors may increase our understanding of potential interventions to minimize tobacco-induced risks to the offspring. Furthermore, identifying potential effect modifiers may support the hypothesized biological mechanisms, which may include structural changes to the developing fetal brain [10] or DNA methylation changes [11].

One such factor is food folate or folic acid [12]. On its own, higher plasma folate during pregnancy is associated with improved attention [13], whereas lower plasma folate is associated with hyperactivity [14] and emotional problems [15]. Folate plays an important role in the synthesis of methionine (a key methyl donor for DNA methylation) [16, 17] and may counteract DNA methylation changes induced by prenatal exposure to tobacco [18].

Similarly, the positive impacts of breastfeeding on offspring neurodevelopment are numerous [19]. Breast milk is rich in nutrients that may confer positive benefits to fetal brain development, such as increased myelination, brain volume, and cortical thickness.[20] Indeed, a longer duration of exclusive breastfeeding has been independently associated with improved cognitive development in early childhood [21]. Yet, these positive attributes may be offset by lactational exposure to tobacco, particularly if the mother is a smoker [22]. Thus, it is unclear whether breastfeeding would mitigate or augment the cognitive and behavioral risks associated with prenatal exposure to tobacco.

We utilized data from *Healthy Start*, a robust cohort of mother-infant pairs living in Colorado followed from the early prenatal period through early childhood, to explore whether there is an interactive effect between prenatal exposure to tobacco and early-life nutrition on offspring cognitive and behavioral health. We hypothesized that higher folate intake during pregnancy and a longer duration of breastfeeding would protect against the adverse effects of prenatal exposure to tobacco on offspring cognitive and behavioral health.

## Methods

*Healthy Start* is a prospective cohort of 1410 ethnically diverse pregnant people who delivered at University of Colorado Hospital. Study participants were pregnant people (≥ 16 years) who were patients at obstetrics clinics at the University of Colorado Hospital (< 24 weeks gestation). Exclusion criteria for study participation included: multiple gestation pregnancies; previous stillbirth or preterm birth at < 25 weeks gestation; preexisting diabetes; asthma; cancer; or a previous psychiatric illness. Participants were invited to participate in two in-person research visits during pregnancy (median: 17 and 27 weeks gestation), one soon after delivery, and one at age 5 years (range: 4–7 years). For our analyses, mother-child pairs were included if the child was born > 37 weeks gestation and > 2500 g at birth.

Written informed consent was obtained from the mother or legal guardian of the child prior to each research visit. Protocols for enrollment and biospecimen collection were approved by the Colorado Multiple Institutional Review Board (#09-0563). The protocol for the current analysis was approved by the University of Texas Health Science Center Committee for the Protection of Human Subjects (HSC-SPH-20-0080).

### Prenatal Exposure to Tobacco

Prenatal exposure to tobacco was assessed via urinary cotinine (major metabolite of nicotine) at ~ 27 weeks gestation. Cotinine was previously analyzed in stored urine samples via solid phase competitive ELISA with a sensitivity of 1 ng/mL (*Calbiotech Cotinine ELISA CO096D, Calbiotech, El Cajon, California*). Consistent with a previous *Healthy Start* analysis [5], cotinine concentrations were dichotomized as no exposure (< 0.05 ng/mL; the limit of detection [LOD]) and prenatal exposure to exposure (> LOD).

### Childhood Cognition

The National Institutes of Health (NIH) Toolbox Cognition Battery is a series of measures used to assess executive function across the lifespan (3–85 years) [23]. A series of tests were administered to children via computer tablets and supervised at each in-person visit by professional research assistants. Measures with the most relevance to our study population included the Flanker test (for assessing inhibitory control), the Dimensional Change Card Sort test (DCCS, for assessing cognitive flexibility), and the Picture Vocabulary Test (for assessing receptive language). Raw scores from these assessments were based on both accuracy and response time (for the Flanker and DCCS) or accuracy (for the picture vocabulary test). Fully corrected T-scores accounted for age, sex, race/ethnicity and maternal education and were standardized to have a mean of 50 and a standard deviation of 10 for all tests [24]. Higher scores reflect better cognitive performance.

### Childhood Behavior

The preschool version of the Child Behavior Checklist (CBCL) (24) was captured at the 5-year visit among *Healthy Start* participants. Parents (or caretakers) were instructed to rate their child’s behaviors and social competencies with responses recorded on a Likert Scale as: 0 = Not True; 1 = Somewhat or Sometimes True; 2 = Very True or Often True. The CBCL scoring system groups behaviors that commonly occurring together as follows: (1) ‘broad band’ composite scales, including externalizing problems (summing scores over rule-breaking and aggressive behaviors), internalizing problems (summing scores for anxious/depressed; withdrawn/depressed; somatic complaints), and a total problems category; (2) empirically-based syndrome scales; and (3) diagnostic and Statistical Manual of Mental Disorders (DSM-IV) oriented scales. Fully corrected T-scores accounted for age and sex and were standardized to have a mean of 50 and a standard deviation of 10 for all tests, with higher scores reflecting more deviant behavior. T-scores ≥ 64 on the composite scales and T-scores ≥ 70 on the syndrome/DSM scales are considered to be in the clinical range for referral to mental health evaluations, with borderline evaluations ranging from 60 to 63 and 65–69 on the composite and syndrome/DSM scales respectively) [25, 26].

### Maternal Folate Intake During Pregnancy

Maternal diet was assessed via the Automated Self-Administered 24-Hour Dietary Recall web-based tool during the pregnancy period. Monthly calls were made throughout the pregnancy (range of 2–8 calls). Individual nutrients were determined using the Nutrition Data System for Research (NDSR) software, at the University of North Carolina at Chapel Hill’s Nutrition and Obesity Research Center. Natural folate and synthetic folic acid from enriched foods were used to determine dietary folate equivalents (DFEs), which account for differences in the absorption of naturally occurring food folate and the more bioavailable synthetic folic acid. Folic acid from dietary supplements was measured by querying brand, type, and dose of supplements used during pregnancy, as previously described [27]. Total maternal folate intake (µg/day) was determined by combining DFE intake (µg/day) and folic acid from supplements (µg/day) in a single folate variable, representing folate from all sources (foods, enriched foods, and supplements). The suggested folate requirement for pregnant people, from food and supplements, is ~ 520 µg/day [28]. Less than 5% of our study population fell below this point—which precluded interaction analyses using this clinical cut-point. Thus, for purposes of this study, maternal intake of folate was assessed using a 25th percentile cut-point (1074 µg/day), a cut-point that has been shown to mitigate the risk of tobacco-induced adverse birth outcomes in previous *Healthy Start* analysis [29].

### Duration of Exclusive Breastfeeding

At the 5 months in-person visit, study participants were asked whether they were: (i) currently feeding their infant any breast milk; (ii) had ever fed their infant formula; or (iii) were currently feeding their infant formula. For this analysis, the duration of exclusive breastfeeding variable was dichotomized as exclusively breastfed from birth to age 5 months (if they answered ‘yes’ to the first question and ‘no’ to the remaining questions) and not exclusively breastfed (if they indicated mixed or formula feeding).

### Covariates

Gestational age was determined based on a mother’s reported date of birth and estimated date of delivery. At enrollment, mothers reported their highest level of education, household income, race, ethnicity, and pre-pregnancy height and weight. At the 5-months visit, mothers were asked to report the number of adults in the household (including themselves) who were regular smokers. Responses to this question ranged from 0 to 6. We dichotomized these data into no household smokers versus any household smokers. Cotinine was measured in childhood urine samples collected at the 5-years follow up visit. Childhood cotinine was dichotomized as no exposure (cotinine < LOD) and any exposure (cotinine ≥ LOD).

### Statistical Analysis

Multivariable linear regression models examined the main effects of maternal folate intake (</≥ 1074 µg/day [the 25th percentile]), breastfeeding duration (</≥5months), and prenatal exposure to tobacco (< LOD, ≥ LOD) on cognitive and behavioral outcomes. Interaction by early-life nutrition was assessed by including a product term between prenatal exposure to tobacco and the dichotomized folate or breastfeeding variables in separate regression models. Confounders were selected based on directed acyclic graphs (DAGS) and previous literature findings. All of our models adjusted for the following covariates: maternal age (years), maternal education (< 12 years; high school degree; any college), maternal race and ethnicity (Hispanic; non-Hispanic Black; non-Hispanic White; all other races and ethnicities combined), postnatal exposure to tobacco (cotinine < LOD, cotinine ≥ LOD), and infant sex. We present adjusted beta coefficients and corresponding 95% confidence intervals (CIs). Statistical significance was set at an alpha level of 0.05 for the main effects models and 0.10 for the interaction models. All statistical analyses were performed using SAS© *OnDemand for Academics*.

## Results

Within the entire *Healthy Start* cohort (n = 1410), the majority of mothers were non-Hispanic white, had household incomes > $70,000, and some college education. These characteristics were similar within the analytic sample for the behavioral outcomes (n = 366). The analytic sample for the cognitive outcomes (n = 189) included mothers who were slightly older, had slightly lower mean daily folate levels, and reported a longer duration of exclusive breastfeeding (results not presented).

Compared to mothers with cotinine levels indicating active smoking or secondhand exposure to tobacco, mothers with no exposure to tobacco were older, had higher household incomes and higher levels of education, consumed more folate during pregnancy, birthed larger babies, breastfed their offspring for a longer duration, and were more likely to be Hispanic or non-Hispanic white (Table 1).

**Table 1:** Characteristics of mother-child pairs according to urinary cotinine in pregnancy by neuro-behavioral sub-samples, Healthy Start (2010–2014)

	NIH toolbox				CBCL			
Mother-child characteristics^b^	Cotinine categories				Cotinine categories			
		< LOD^a^	≥ LOD			< LOD^a^	≥ LOD	
	Total (n=189)	No exposure (n=144)	Any smoking exposure (n=45)	p-value^e^	Total^f^ (n=366)	No exposure (n=288)	Any smoking exposure (n=78)	p-value^e^
Maternal characteristics^c^								
Maternal age (yrs)	30 ± 6	30 ± 5	26 ± 7	< 0.01	29 ± 6	30 ± 5	25 ± 6	< 0.01
Pre-pregnancy BMI (kg/m2)	25 ± 5	25 ± 5	27 ± 7	< 0.01	26 ± 6	26 ± 6	27 ± 8	0.03
Gravidity, number of pregnancies	1 ± 1	1 ± 1	1 ± 1	0.28	1 ± 1	1 ± 1	1 ± 1	0.63
Maternal race/ethnicity								
Non-hispanic white	131 (69%)	113 (78%)	18 (40%)	< 0.01	220 (60%)	190 (66%)	30 (38%)	< 0.01
Non-hispanic black	20 (11%)	6 (4%)	14 (31%)		34 (9%)	11 (4%)	23 (29%)	
Hispanic	28 (15%)	19 (13%)	9 (20%)		93 (25%)	76 (26%)	17 (22%)	
Other	10 (5%)	6 (4%)	4 (9%)		19 (5%)	11 (4%)	8 (10%)	
Household income								
<40,000	35 (19%)	18 (12%)	17 (38%)	< 0.01	83 (23%)	55 (19%)	28 (36%)	< 0.01
40,001–70,000	32 (17%)	25 (17%)	7 (16%)		67 (18%)	53 (18%)	14 (18%)	
>70,000	100 (53%)	93 (65%)	7 (16%)		148 (40%)	139 (48%)	9 (11%)	
Don't know	22 (12%)	8 (6%)	14 (31%)		68 (19%)	41 (14%)	27 (35%)	
Mother's highest level of education								
<12 years	12 (6%)	2 (1%)	10 (22%)	< 0.01	42 (11%)	23 (8%)	19 (24%)	< 0.01
High school degree	24 (13%)	14 (10%)	10 (22%)		54 (15%)	33 (12%)	21 (27%)	
College classes or college degree	153 (81%)	128 (89%)	25 (56%)		270 (74%)	232 (80%)	38 (49%)	
Total prenatal folic acid supplementation & dietary folate equivalents (µg/day)^d^	1352 ± 444	1389 ± 419	1233 ± 444	0.04	1384 ± 567	1420 ± 574	1247 ± 529	0.02
Total folic acid supplementation & dietary folate equivalents^d^ [1074µg/day=25th percentile]								
<1074	49 (26%)	30 (21%)	19 (42%)	< 0.01	95 (26%)	64 (22%)	31 (40%)	< 0.01
≥1074	139 (74%)	1113 (78%)	26 (58%)		269 (74%)	222 (77%)	47 (60%)	
Missing	1 (0%)	1 (1%)	0 (0%)		2 (0%)	2 (1%)	0 (0%)	
Duration of exclusive breastfeeding (mo)								
<5	81 (43%)	51 (35%)	30 (67%)	< 0.01	171 (47%)	119 (41%)	52 (67%)	< 0.01
≥5	97 (51%)	86 (60%)	11 (24%)		166 (45%)	150 (52%)	16 (21%)	
Missing	11 (6%)	7 (5%)	4 (9%)		29 (8%)	19 (7%)	10 (13%)	
Child characteristics								
Male	92 (49%)	69 (48%)	23 (51%)	0.71	185 (50%)	148 (51%)	37 (47%)	0.44
Birth weight (g)	3336 ± 411	3358 ± 415	3265 ± 395	0.19	3317 ± 394	3348 ± 396	3202 ± 367	< 0.01
Gestational age at birth (weeks)	40 ± 1	40 ± 1	40 ± 1	0.71	40 ± 1	40 ± 1	40 ± 1	0.59
Childhood BMI at 5 yr visit (kg/m^2^)	16 ± 7	16 ± 8	16 ± 1	0.59	16 ± 2	16 ± 2	16 ± 2	0.55
Approximate age at outcome assessment (yrs)	5 ± 0.3	5 ± 0.3	5 ± 0.2	0.78	5 ± 0.4	5 ± 0.4	5 ± 0.3	0.17

Continuous variables shown as mean ± standard deviations; categorical variables displayed as proportions of column totals

*LOD* limit of detection; *CBCL* the child behavior checklist; *ASQ-3* the ages and stages questionnaire; *NIH* National Institutes of Health; *mo* months

^a^Cotinine levels expressed in nanograms/milliliter (ng/ml); limit of detection (LOD)= ~0.05 ng/ml

^b^Mother-child pairs excluded from all analyses included: mothers missing cotinine data; mothers diagnosed with a previous psychiatric condition; preterm births (< 37 weeks gestation); low birthweight infants (< 2500g); participants missing data for each of the respective neuro-behavioral outcome tools assessed

^c^All maternal characteristics, unless otherwise noted, were assessed at 17 weeks pregnancy visit

^d^Dietary characteristics collected using the automated self-administered 24-hour dietary recall (ASA24) at minimum twice over the course of the pregnancy (range: 2–8 times)

^e^Independent samples t-tests used to assess differences in means across cotinine categories for continuous variables (means +/− standard deviations). Chi-square square tests used to examine proportion differences across urinary cotinine categories

^f^Children administered the CBCL > 5 years old also excluded from all analyses

Our main effects models revealed that prenatal exposure to tobacco was moderately associated with decreased inhibitory control (beta coefficient: − 3.2; 95% CI: − 6.8, 0.3; p = 0.07), lower maternal folate intakes were associated with higher anxious/depressed T-scores (beta coefficient: 1.1; 95% CI: 0.1, 2.1; p = 0.04), and a shorter duration of breastfeeding (< 5 months) was associated with lower receptive language scores (beta coefficient: − 4.3; 95% CI: − 8.5, 0.0; p = 0.05) (Table 2).

**Table 2:** Adjusted beta coefficients for maternal prenatal folate intake, breastfeeding duration, and prenatal cotinine categories and selected neuro-cognitive and behavioral outcomes, *Healthy Start* (2010–2014)

Neuro-cognitive or behavioral tool (assessed at 5-year visit)	Prenatal cotinine (≥LOD vs. <LOD (ref))^a^	Breastfeeding (<5 vs. ≥5 months (ref))^a^	Maternal folate intake (<1074µg/day vs. ≥1074µg/day (ref))^a^
NIH toolbox^b^			
Flanker	− 3.24 (− 6.80, 0.32); p = 0.07	− 2.06 (− 4.62, 0.50); p = 0.11	− 0.56 (− 3.23, 2.12); p = 0.68
Dimensional change card sort test	− 0.37 (− 5.10, 4.36); p = 0.88	− 1.44 (− 4.84, 1.96); p = 0.40	2.12 (− 1.43, 5.66); p = 0.24
Picture vocabulary	− 0.73 (− 6.61, 5.14); p = 0.81	− 4.25 (− 8.48, − 0.02); p = 0.05	0.54 (− 3.88, 4.95); p = 0.81
CBCL composite scales^c^			
Externalziing	− 0.10 (− 3.54, 3.33); p = 0.95	− 1.36 (− 3.75, 1.03); p = 0.26	− 0.45 (− 3.01, 2.11); p = 0.73
Internalizing	− 1.22 (− 4.82, 2.39); p = 0.51	− 0.28 (− 2.79, 2.23); p = 0.83	1.15 (− 1.54, 3.83); p = 0.40
Total	− 0.69 (− 4.18, 2.81); p = 0.70	− 0.22 (− 2.66, 2.21); p = 0.86	− 0.11 (− 2.72, 2.49); p = 0.93
CBCL—syndrome scalesc			
Anxious/depressed	− 0.60 (− 1.98, 0.77); p = 0.39	− 0.27 (− 1.23, 0.68); p = 0.57	1.08 (0.06, 2.10); p = 0.04
Withdrawn	− 0.65 (− 2.43, 1.14); p = 0.48	0.72 (− 0.53, 1.96); p = 0.26	− 0.15 (− 1.48, 1.18); p = 0.82
Somatic complaints	0.43 (− 1.29, 2.15); p = 0.62	0.07 (− 1.12, 1.27); p = 0.91	0.03 (− 1.24, 1.31); p = 0.96
Attention problems	0.33 (− 1.32, 1.99); p = 0.69	0.54 (− 0.61, 1.68); p = 0.36	0.03 (− 1.20, 1.26); p = 0.96
Emotionally reactive	− 0.26 (− 2.10, 1.57); p = 0.78	− 0.82 (− 2.09, 0.45); p = 0.21	0.64 (− 0.73, 2.00); p = 0.36
Sleep problems	− 0.15 (− 2.03, 1.74); p = 0.88	− 0.63 (− 1.94, 0.69); p = 0.35	− 0.36 (− 1.77, 1.04); p = 0.61
Aggressive behaviors	− 0.35 (− 1.84, 1.13); p = 0.64	− 0.70 (− 1.73, 0.33); p = 0.18	− 0.06 (− 1.17, 1.04); p = 0.91
CBCL—DSM (IV)-oriented scalesc			
Anxiety problems	− 0.01 (− 1.66, 1.65); p = 0.99	− 0.23 (− 1.38, 0.92); p = 0.69	0.57 (− 0.67, 1.80); p = 0.37
ADHD	− 0.24 (− 1.75, 1.26); p = 0.75	0.04 (− 1.01, 1.09); p = 0.94	0.08 (− 1.04, 1.21); p = 0.88
Oppositional defiant	− 0.44 (− 2.05, 1.17); p = 0.59	− 0.73 (− 1.85, 0.39); p = 0.20	0.37 (− 0.82, 1.57); p = 0.54
Autism spectrum	− 0.54 (− 2.27, 1.19); p = 0.54	0.56 (− 0.64, 1.77); p = 0.36	− 1.13 (− 2.42, 0.16); p = 0.09
Depressive problems	− 0.01 (− 1.74, 1.74); p = 0.99	− 0.09 (− 1.30, 1.13); p = 0.89	− 0.33 (− 1.63, 0.97); p = 0.62

*NIH Toolbox* National Institutes of Health Toolbox {Cognition Battery Tool utilized within this study}; *CBCL* child behavior checklist {preschool edition, assessed at age 5}; *ADHD* attention-deficit/hyperactivity disorder; *DSM* diagnostic and statistcal manual of mental Ddisorders; Adj. Beta Coefficients/95%CIs=Adjusted Beta Coefficients/95% Confidence Intervals; *LOD* limit of detection

^a^All models adjusted for maternal age (years), education (< high school, high school diploma, some college), race/ethnicity (non-Hispanic (NH) white; NH black, Hispanic, other), previous diagnosis of a psychiatric condition, offspring sex, household reported smokers at 5 months (any; none), cotinine levels detected at 5 yrs (any; none), and each of the other respective effect modifiers (e.g. maternal folate models were also adjusted for breastfeeding duration and prenatal cotinine)

^b^Higher NIH Toolbox T-scores indicative of *better* performanace on each of the cognition tests administered

^c^Higher CBCL T-scores indicative of *worse* performanace on each of the cognition tests administered

The interaction results indicate that the duration of breastfeeding modified the association between prenatal exposure to tobacco with sleep problems (p for interaction = 0.01), depressive problems (p for interaction = 0.02), and total problems (p for interaction = 0.09) (Table 3) To

**Table 3:** Adjusted beta coefficients for maternal cotinine categories and fully adjusted T-Scores for selected cognitive and neuro-cognitive and behavioral outcomes by prenatal daily folate intake and breastfeeding duration, *Healthy Start *(2010–2014**)**

	Adjusted mean differences for fetal exposure to tobacco within strata of prenatal folate intake^a^					Adjusted mean differences for fetal exposure to tobacco within strata of breastfeeding^b^				
Cognitive or behavioral tool (assessed at 5-year visit)	< 1074µg/day		≥ 1074µg/day			< 5 months		≥ 5 months		
	n_1_/n_0_^c^	Mean difference (95% CI)	n_1_/n_0_^c^	Mean difference (95% CI)	p for interaction^d^	n_1_/n_0_^c^	Mean difference (95% CI)	n_1_/n_0_^c^	Mean difference (95% CI)	p for interaction^d^
NIH toolbox										
Inhibitory control	30/19	− 2.4 (− 8.0, 3.2)	113/26	− 4.7 (− 8.7, − 0.7)	1.0 (− 4.8, 6.9); p = 0.65	50/29	− 5.3 (− 12.0, 1.4)	81/11	− 1.6 (− 5.6, 2.5)	3.7 (− 2.8, 10.2); p = 0.18
Cognitive flexibility		0.2 (− 6.6, 6.9)		0.2 (− 5.4, 5.7)	− 0.5 (− 7.9, 7.0); p = 0.97		0.8 (− 6.9, 8.4)		− 1.9 (− 9.1, 5.4)	0.0 (− 8.6, 8.5); p = 0.87
Receptive language		− 2.3 (− 12.8, 8.3)		1.0 (− 5.7, 7.6)	0.6 (− 9.2, 10.4); p = 0.66		2.2 (− 8.3, 12.7)		− 4.5 (− 12.4, 3.4)	− 5.3 (− 14.8, 5.8); p = 0.39
CBCL: broadband scales										
Total problems	64/31	− 1.6 (− 6.9, 3.7)	222/47	0.5 (− 3.5, 4.6)	3.6 (− 2.0, 9.3); p = 0.19	105/48	− 1.9 (− 6.9, 3.0)	130/14	1.3 (− 4.2, 6.8)	5.8 (− 0.7, 12.4); p = 0.09
Internalizing		− 2.2 (− 7.6, 3.1)		− 0.5 (− 4.8, 3.8)	3.4 (− 2.4, 9.3); p = 0.22		− 2.3 (− 7.2, 2.6)		0.9 (− 5.2, 6.9)	5.7 (− 1.0, 12.5); p = 0.12
Externalizing		− 0.7 (− 6.2, 4.9)		0.6 (− 3.3, 4.5)	2.9 (− 2.7, 8.4); p = 0.33		− 0.5 (− 5.3, 4.3)		0.3 (− 5.2, 5.8)	3.3 (− 3.1, 9.8); p = 0.26
CBCL: syndrome scales										
Anxious/depressed	64/31	− 0.4 (-− 3.0, 2.2)	222/47	− 1.3 (− 2.9, 0.4)	0.4 (− 2.0, 2.7); p = 0.84	105/48	− 0.1 (− 2.1, 1.9)	130/14	− 0.8 (− 3.0, 1.5)	0.6 (− 2.0, 3.3); p = 0.65
Withdrawn		− 2.1 (− 4.8, 0.6)		0.0 (-2.2, 2.2)	1.5 (− 1.5, 4.6); p = 0.30		− 0.8 (− 3.6, 1.9)		− 0.3 (− 2.9, 2.2)	1.1 (− 2.2, 4.5); p = 0.55
Somatic complaints		− 0.6 (− 3.2, 2.0)		1.0 (-1.0, 3.0)	1.8 (− 1.0, 4.6); p = 0.16		− 0.6 (− 2.9, 1.8)		1.8 (− 1.1, 4.7)	2.9 (0.4, 6.1); p = 0.10
Attention problems		− 0.5 (− 3.3, 2.3)		0.8 (− 1.1, 2.6)	− 1.6 (− 1.1, 4.3); p = 0.38		1.1 (− 1.5, 3.7)		− 0.6 (− 2.7, 1.6)	0.1 (− 3.1, 3.2); p = 0.93
Emotionally reactive		1.1 (− 2.0, 4.3)		− 1.6 (− 3.7, 0.4)	− 1.3 (− 4.3, 1.7); p = 0.32		0.1 (− 2.3, 2.4)		− 0.6 (− 3.9, 2.6)	0.4 (− 3.1, 3.8); p = 0.76
Sleep problems		− 1.3 (− 4.4, 1.8)		1.6 (− 0.6, 3.7)	1.6 (− 1.4, 4.7); p = 0.25		− 0.7 (− 3.2, 1.8)		1.7 (− 1.6, 5.0)	4.6 (1.0, 8.2); p = 0.01
Aggressive behaviors		− 0.3 (− 2.7, 2.1)		0.1 (− 1.6, 1.7)	0.9 (− 1.5, 3.3); p = 0.36		0.1 (− 1.9, 2.1)		− 0.4 (− 2.9, 2.0)	0.9 (− 1.9, 3.6); p = 0.43
CBCL: DSM-oriented scales										
Anxiety problems	64/31	− 0.5 (− 3.43, 2.5)	222/47	0.2 (− 1.8, 2.3)	1.6 (− 1.2, 4.5); p = 0.40	105/48	0.2 (− 2.1, 2.4)	130/14	0.1 (− 2.5, 2.7)	1.1 (− 2.1, 4.2); p = 0.58
ADHD		− 0.3 (− 2.90, 2.3)		− 0.1 (− 1.7, 1.5)	1.0 (− 1.4, 3.5); p = 0.41		1.3 (− 1.0, 3.5)		− 1.8 (− 3.8, 0.2)	− 0.7 (− 3.5, 2.2); p = 0.67
Oppositional defiant		− 0.3 (− 3.13, 2.5)		0.0 (− 1.8, 1.7)	0.5 (− 2.1, 3.1); p = 0.47		− 0.3 (− 2.4, 1.9)		− 0.2 (− 2.8, 2.5)	1.5 (− 1.5, 4.5); p = 0.26
Autism spectrum		− 0.5 (− 2.8, 1.8)		− 0.3 (− 2.5, 1.8)	1.3 (− 1.6, 4.2); p = 0.50		− 0.5 (− 3.0, 2.0)		− 0.5 (− 3.3, 2.2)	1.1 (− 2.2, 4.3); p = 0.63
Stress problems		− 0.9 (− 4.1, 2.4)		0.0 (− 2.0, 1.9)	1.5 (− 1.5, 4.5); p = 0.34		0.7 (− 2.1, 3.4)		− 0.2 (− 2.8, 2.3)	0.9 (− 2.5, 4.3); p = 0.51
Depressive problems		− 0.4 (− 3.0, 2.2)		0.2 (− 2.1, 2.5)	0.6 (− 2.2, 3.4); p = 0.70		0.2 (− 2.1, 2.5)		2.1 (− 0.7, 5.0)	3.8 (0.5, 7.1); p = 0.02

Missings for factors controlled for across all models were excluded from totals

*NIH Toolbox* National Institutes of Health Toolbox {Cognition Battery Tool utilized within this study}; *CBCL* child behavior checklist {preschool edition, assessed at age 5}; *ADHD* attention-deficit/hyperactivity disorder; *DSM* diagnostic and statistcal manual of mental disorders; *Adj. Beta coefficients/95%CIs* adjusted beta coefficients/95% confidence intervals

^a^All models adjusted for maternal age (years), education (< high school, high school diploma, some college), race/ethnicity (non-Hispanic (NH) white; NH black, Hispanic, other), previous diagnosis of a psychiatric condition, offspring sex and and cotinine levels detected at 5 yrs (any; none).

^b^All models adjusted for maternal age (years), education (< high school, high school diploma, some college), race/ethnicity (non-Hispanic (NH) white; NH black, Hispanic, other), previous diagnosis of a psychiatric condition, offspring sex, household reported smokers at 5 months (any; none), and cotinine levels detected at 5 yrs (any; none).

^e^(n_1/_n_0_)=Exposed to SHS/Not-exposed; n’s shown at top of each category are the same across each of the other respective outcomes within each group (e.g. 30/19 is the same sample size for DCCS and picture vocabulary tests within the NIH toolbox, < 1074µg folate/day group)

^d^p-values for interaction generated by adding product terms between maternal cotinine and folate (< 1074; ≥ 1074µg/day) or breastfeeding duration (<5; ≥5 months) in separate models.

bacco-exposed offspring had more sleep problems (beta: 3.8; 95% CI: 0.5, 7.1; interaction p-value = 0.02), depressive problems (beta: 4.6; 95% CI: 1.0, 8.2; interaction p-value = 0.01), and total problems (beta: 5.8; 95% CI: − 0.7, 12.4; interaction p-value = 0.09) if they were exclusively breastfed for at least 5 months, but not for shorter durations. We found no evidence of an interaction between prenatal exposure to tobacco and folate with any of the other behavioral or cognitive outcomes.

## Discussion

In this exploratory analysis, we found some indications that a shorter duration of breastfeeding, lower maternal folate intakes, and prenatal exposure to tobacco were associated with adverse cognitive and behavioral traits in early childhood. Our interaction results further revealed that the combination of prenatal exposure to tobacco and a longer duration of breastfeeding was associated with more behavioral problems in early childhood, which may be due to lactational exposure to tobacco byproduct. This key finding supports the need for smoking cessation efforts beginning in pregnancy and throughout postpartum period (when mothers are encouraged to breastfeed) to minimize neurobehavioral risks to the offspring. These preliminary findings should be followed up in larger prospective cohorts.

A shorter duration of breastfeeding was associated with decreased receptive language, which mirrors findings by Oddy and colleagues [30]. Yet, we found no other evidence that exclusive breastfeeding influenced any of the other cognitive or behavioral outcomes. This may be somewhat expected, given that a recent meta-analysis of 80 epidemiologic studies described how many of the positive effects of breastfeeding on childhood neurodevelopment disappeared after controlling for confounders, such as maternal cognitive and socioeconomic status [31].

The American Academy of Pediatrics’ recommends that infants be exclusively breastfed for at least six months, regardless of tobacco use or exposure [32]. Consistent with this recommendation, we hypothesized that a longer duration of breastfeeding would mitigate the effects of prenatal exposure to tobacco on adverse cognitive and behavioral outcomes, despite potential lactational exposure to tobacco byproducts. Yet, we found the opposite: a longer period of breastfeeding combined with prenatal exposure to tobacco was associated with more sleep problems, depressive problems, and total problems in early childhood. How these exposures, which occur in the fetal and early postnatal periods, influence sleep at age 5 years is not clear. Offspring with prenatal exposure to tobacco may also experience postnatal exposure to tobacco, particularly if the mother is a smoker. Greater doses of nicotine delivered to infants via breastmilk appeared to disrupt infant sleep cycles [33], which may be a result of reduced breast milk supply [34], changes in breast milk composition [35], or the stimulating effects of nicotine [36]. Sleep problems in early infancy may persist for many years [37]. These sleep disruptions may ultimately contribute to behavior problems [38] and depressive symptoms [39] in childhood, which further supports our finding that prenatal exposure to tobacco and a longer duration of breastfeeding was associated with more problem behaviors overall.

A majority (95%) of the pregnant people in our study had folate levels adequate for minimizing neural tube defect risk (> 520 µg/day). Even so, higher folate intakes during pregnancy were associated with increased anxious/depressed symptoms in the 5-year-old offspring. However, contrary to other observational studies [13, 14] and a recent clinical trial [40], folate intake did not appear to influence child cognition or other behavioral outcomes. It is important to note that we excluded offspring born prior to 37 weeks, as even moderate preterm birth (32–37 weeks) increases the risk for developmental delays [41]. Only one other study excluded these high-risk offspring and reported no association between folate and infant cognitive development [42]. Furthermore, our null findings could be partially attributed to folate intake being determined via self-report of diet and supplements (whereas previously published studies have used biomarkers to determine folate status [13, 14, 40]), as well as our small sample (n = 189), which may have limited our ability to detect subtle changes in child cognition and behavior.

Furthermore, we found no evidence of an interaction between folate intake and tobacco on childhood cognition or behavior, which may point to distinct and unrelated biological mechanisms. For instance, folate may impact childhood cognition and behavior via homocysteine pathways [43, 44], whereas prenatal exposure to tobacco may contribute to adverse cognitive and behavioral traits in children via overstimulation of fetal nicotinic acetylcholine receptors [45] or tobacco-induced fetal hypoxia [46], resulting in long-lasting detriments to brain morphology [10]. Whether epigenetics play a role warrants further investigation [11].

Our study may be limited by the neurobehavioral measures used to assess childhood cognition and behavior. Although the CBCL is being increasingly used in clinical settings, it may fail to identify certain mental disorders [47] or capture ‘episodicity’ (i.e. the occurrence of sporadic or irregular events) [48], which are common among children with certain behavioral problems [49]. Similarly, the validity of the NIH toolbox among younger children (ages 3–6 years) has been difficult to assess due to the absence of a gold standard for targeted constructs for these ages.

An important limitation of our approach is inability to determine lactational exposures to tobacco in breast milk. Because *Healthy Start* did not collect breast milk samples, we lack data on chemical or nutritional composition of breast milk. Furthermore, mothers were not explicitly asked about their tobacco use or exposure while breastfeeding. Similarly, we lack data on diet and supplements during the periconceptional period (three months prior to conception through the second month of pregnancy). This represents a critical developmental window in which folate may have a profound effect on child neurodevelopment [50] and substantially lower autism risk [51, 52]. Thus, folate intake during this period may have the greatest benefit for reducing the cognitive and behavioral burden of tobacco-exposed. Future prospective studies are needed to explore this important area of research.

Small numbers in analytic samples—particularly in the examinations of low folate and tobacco-exposed groups—may have hindered our ability to detect meaningful associations or interactions in some of our outcome groups. Even so, we cannot rule out the potential for chance findings given that we performed 54 separate main effects analyses and 38 separate interaction analyses. Correction for multiple testing (e.g. Bonferroni set to p < 0.003 = 0.10 / 38 for the interaction analyses) would impose a severe penalty on the results, many of which would drop out of statistical significance.

One of the primary strengths of this study included the use of cotinine to objectively measure tobacco exposure both during the prenatal and postnatal periods. Second, the use of the ASA24 online platform to capture total daily folate intake (which was utilized at multiple time points throughout pregnancy) reduced the potential for reporting errors and recall bias in our examinations. Lastly, following mother-child pairs from the prenatal period through the first few years of life (for the participating children) is a unique strength of *Healthy Start*—providing insight into little-studied associations between the early fetal period and later neurobehavioral outcomes with important preventative care implications.

## Summary

Prenatal exposure to tobacco is associated with adverse cognitive and behavioral traits in childhood [2–5]. Certain nutritional factors, such as folate intake during pregnancy or breastfeeding, may modify the adverse effects of prenatal exposure to tobacco. However, since breast milk may contain tobacco byproducts [22], it is unclear whether breastfeeding would offset or augment the cognitive and behavioral risks induced by prenatal exposure to tobacco. In this exploratory analysis, we examined whether higher intakes of folate during pregnancy or a longer duration of exclusive breastfeeding modified the associations between prenatal exposure to tobacco and childhood cognition/behavior. The combination of prenatal exposure to tobacco and a longer duration of exclusive breastfeeding was associated with more sleep problems, depressive problems, and total problems in early childhood. While there are many public health benefits to longer durations of breastfeeding (e.g. reduced risk for obesity) [53], it remains important to encourage pregnant people to quit smoking and make efforts to avoid secondhand exposure to tobacco to limit adverse cognitive and behavioral outcomes in the offspring.

## References

[1] Shiohama T, Hisada A, Yamamoto M et al (2021) Decreased head circumference at birth associated with maternal tobacco smoke exposure during pregnancy on the japanese prospective birth cohort study. Sci Rep 11(1):18949 34556740 10.1038/s41598-021-98311-2 PMC8460647

[2] Polańska K, Jurewicz J, Hanke W (2015) Smoking and alcohol drinking during pregnancy as the risk factors for poor child neurodevelopment - A review of epidemiological studies. Int J Occup Med Environ Health 28(3):419–443 26190723 10.13075/ijomeh.1896.00424

[3] Eskenazi B, Castorina R (1999) Association of prenatal maternal or postnatal child environmental tobacco smoke exposure and neurodevelopmental and behavioral problems in children. Environ Health Perspect 107(12):991–1000 10585903 10.1289/ehp.99107991 PMC1566803

[4] Herrmann M, King K, Weitzman M (2008) Prenatal tobacco smoke and postnatal secondhand smoke exposure and child neurodevelopment. Curr Opin Pediatr 20(2):184–190 18332716 10.1097/MOP.0b013e3282f56165

[5] Moore BF, Shapiro AL, Wilkening G et al (2020) Prenatal exposure to Tobacco and offspring neurocognitive development in the healthy start study. J Pediatr 218:28-34e22 31759580 10.1016/j.jpeds.2019.10.056 PMC7042047

[6] Levin ED, Briggs SJ, Christopher NC, Rose JE (1993) Prenatal nicotine exposure and cognitive performance in rats. Neurotoxicol Teratol 15(4):251–260 8413079 10.1016/0892-0362(93)90006-a

[7] Johns JM, Louis TM, Becker RF, Means LW (1982) Behavioral effects of prenatal exposure to nicotine in guinea pigs. Neurobehav Toxicol Teratol 4(3):365–369 7099355

[8] Yanai J, Pick CG, Rogel-Fuchs Y, Zahalka EA (1992) Alterations in hippocampal cholinergic receptors and hippocampal behaviors after early exposure to nicotine. Brain Res Bull 29(3–4):363–368 1393609 10.1016/0361-9230(92)90069-a

[9] Tsai J, Homa DM, Gentzke AS et al (2018) Exposure to secondhand smoke among nonsmokers - United States, 1988–2014. MMWR Morb Mortal Wkly Rep 67(48):1342–1346 30521502 10.15585/mmwr.mm6748a3 PMC6329485

[10] El Marroun H, Schmidt MN, Franken IH et al (2014) Prenatal tobacco exposure and brain morphology: a prospective study in young children. Neuropsychopharmacology 39(4):792–800 24096296 10.1038/npp.2013.273 PMC3924517

[11] Chatterton Z, Hartley BJ, Seok MH et al (2017) In utero exposure to maternal smoking is associated with DNA methylation alterations and reduced neuronal content in the developing fetal brain. Epigenetics Chromatin 10:4 28149327 10.1186/s13072-017-0111-y PMC5270321

[12] Iyer R, Tomar SK (2009) Folate: a functional food constituent. J Food Sci 74(9):R114–122 20492126 10.1111/j.1750-3841.2009.01359.x

[13] Veena SR, Krishnaveni GV, Srinivasan K et al (2010) Higher maternal plasma folate but not vitamin B-12 concentrations during pregnancy are associated with better cognitive function scores in 9- to 10- year-old children in South India. J Nutr 140(5):1014–1022 20335637 10.3945/jn.109.118075 PMC3672847

[14] Schlotz W, Jones A, Phillips DI, Gale CR, Robinson SM, Godfrey KM (2010) Lower maternal folate status in early pregnancy is associated with childhood hyperactivity and peer problems in offspring. J Child Psychol Psychiatry 51(5):594–602 19874428 10.1111/j.1469-7610.2009.02182.x PMC2862762

[15] Steenweg-de Graaff J, Roza SJ, Steegers EA et al (2012) Maternal folate status in early pregnancy and child emotional and behavioral problems: the generation R study. Am J Clin Nutr 95(6):1413–1421 22572645 10.3945/ajcn.111.030791

[16] Steegers-Theunissen RP, Smith SC, Steegers EA, Guilbert LJ, Baker PN (2000) Folate affects apoptosis in human trophoblastic cells. BJOG 107(12):1513–1515 11192109 10.1111/j.1471-0528.2000.tb11677.x

[17] Bakker R, Timmermans S, Steegers EA, Hofman A, Jaddoe VW (2011) Folic acid supplements modify the adverse effects of maternal smoking on fetal growth and neonatal complications. J Nutr 141(12):2172–2179 22031658 10.3945/jn.111.142976

[18] Zhang B, Hong X, Ji H et al (2018) Maternal smoking during pregnancy and cord blood DNA methylation: new insight on sex differences and effect modification by maternal folate levels. Epigenetics 13(5):505–518 29945474 10.1080/15592294.2018.1475978 PMC6140808

[19] Krol KM, Grossmann T (2018) Psychological effects of breastfeeding on children and mothers. Bundesgesundheitsblatt - Gesundheitsforschung - Gesundheitsschutz 61(8):977–985 29934681 10.1007/s00103-018-2769-0 PMC6096620

[20] Deoni S, Dean D III, Joelson S, O’Regan J, Schneider N (2018) Early nutrition influences developmental myelination and cognition in infants and young children. NeuroImage 178:649–659 29277402 10.1016/j.neuroimage.2017.12.056 PMC6540800

[21] Lee H, Park H, Ha E et al (2016) Effect of breastfeeding duration on cognitive development in infants: 3-year follow-up study. J Korean Med Sci 31(4):579–584 27051242 10.3346/jkms.2016.31.4.579 PMC4810341

[22] Rowe H, Baker T, Hale TW (2015) Maternal medication, drug use, and breastfeeding. Child Adolesc Psychiatr Clin N Am 24(1):1–20 25455573 10.1016/j.chc.2014.09.005

[23] Weintraub S, Dikmen SS, Heaton RK et al (2013) Cognition assessment using the NIH toolbox. Neurology 80(11 Suppl 3):S54-64 23479546 10.1212/WNL.0b013e3182872ded PMC3662346

[24] Casaletto KB, Umlauf A, Beaumont J et al (2015) Demographically corrected normative standards for the English version of the NIH toolbox cognition battery. J Int Neuropsychol Soc 21(5):378–391 26030001 10.1017/S1355617715000351 PMC4490030

[25] Achenbach TM, Becker A, Döpfner M et al (2008) Multicultural assessment of child and adolescent psychopathology with ASEBA and SDQ instruments: research findings, applications, and future directions. J Child Psychol Psychiatry 49(3):251–275 18333930 10.1111/j.1469-7610.2007.01867.x

[26] Biederman J, DiSalvo M, Vaudreuil C et al (2020) Can the child behavior checklist (CBCL) help characterize the types of psychopathologic conditions driving child psychiatry referrals? Scand J Child Adolesc Psychiatr Psychol 8:157–165 33564632 10.21307/sjcapp-2020-016 PMC7866779

[27] Sauder KA, Harte RN, Ringham BM et al (2021) Disparities in risks of inadequate and excessive intake of micronutrients during pregnancy. J Nutr 151(11):3555–3569 34494118 10.1093/jn/nxab273 PMC8564697

[28] Press NA (1998) Dietary reference intakes for thiamin, riboflavin, niacin, vitamin B6, folate, vitamin B12, pantothenic acid, biotin, and choline1. Institute of Medicine 23193625

[29] Hoyt AT, Wilkinson AV, Langlois PH et al (2022) Prenatal exposure to tobacco and adverse birth outcomes: effect modification by folate intake during pregnancy. Matern Health Neonatol Perinatol 8(1):6 36096906 10.1186/s40748-022-00141-1 PMC9465971

[30] Oddy WH, Kendall GE, Blair E et al (2003) Breast feeding and cognitive development in childhood: a prospective birth cohort study. Paediatr Perinat Epidemiol 17(1):81–90 12562475 10.1046/j.1365-3016.2003.00464.x

[31] Walfisch A, Sermer C, Cressman A, Koren G (2013) Breast milk and cognitive development–the role of confounders: a systematic review. BMJ Open 3(8):e003259 23975102 10.1136/bmjopen-2013-003259 PMC3753522

[32] Transfer of drugs (2001) And other chemicals into human milk. Pediatrics 108(3):776–789 11533352 10.1542/peds.108.3.776

[33] Mennella JA, Yourshaw LM, Morgan LK (2007) Breastfeeding and smoking: short-term effects on infant feeding and sleep. Pediatrics 120(3):497–502 17766521 10.1542/peds.2007-0488 PMC2277470

[34] Bahadori B, Riediger ND, Farrell SM, Uitz E, Moghadasian MF (2013) Hypothesis: smoking decreases breast feeding duration by suppressing prolactin secretion. Med Hypotheses 81(4):582–586 23948597 10.1016/j.mehy.2013.07.007

[35] Hopkinson JM, Schanler RJ, Fraley JK, Garza C (1992) Milk production by mothers of premature infants: influence of cigarette smoking. Pediatrics 90(6):934–938 1437437

[36] Vazquez J, Guzmán-Marín R, Salín-Pascual RJ, Drucker-Colín R (1996) Transdermal nicotine on sleep and PGO spikes. Brain Res 737(1–2):317–320 8930383 10.1016/0006-8993(96)00921-3

[37] Kataria S, Swanson MS, Trevathan GE (1987) Persistence of sleep disturbances in preschool children. J Pediatr 110(4):642–646 3559818 10.1016/s0022-3476(87)80571-1

[38] Lavigne JV, Arend R, Rosenbaum D et al (1999) Sleep and behavior problems among preschoolers. J Dev Behav Pediatr 20(3):164–169 10393073 10.1097/00004703-199906000-00005

[39] Rao U (2011) Sleep disturbances in pediatric depression. Asian J Psychiatr 4(4):234–247 22287998 10.1016/j.ajp.2011.09.001 PMC3265574

[40] Caffrey A, McNulty H, Rollins M et al (2021) Effects of maternal folic acid supplementation during the second and third trimesters of pregnancy on neurocognitive development in the child: an 11-year follow-up from a randomised controlled trial. BMC Med 19(1):73 33750355 10.1186/s12916-021-01914-9 PMC7945668

[41] Kerstjens JM, Bocca-Tjeertes IF, de Winter AF, Reijneveld SA, Bos AF (2012) Neonatal morbidities and developmental delay in moderately preterm-born children. Pediatrics 130(2):e265–272 22778308 10.1542/peds.2012-0079

[42] Wu BT, Dyer RA, King DJ, Richardson KJ, Innis SM (2012) Early second trimester maternal plasma choline and betaine are related to measures of early cognitive development in term infants. PLoS ONE 7(8):e43448 22916264 10.1371/journal.pone.0043448 PMC3423345

[43] Black MM (2008) Effects of vitamin B12 and folate deficiency on brain development in children. Food Nutr Bull 29(2 Suppl):S126–131 18709887 10.1177/15648265080292S117 PMC3137939

[44] Ars CL, Nijs IM, Marroun HE et al (2019) Prenatal folate, homocysteine and vitamin B(12) levels and child brain volumes, cognitive development and psychological functioning: the generation R study. Br J Nutr 122(s1):S1-s9 31638501 10.1017/S0007114515002081

[45] Slotkin TA, Southard MC, Adam SJ, Cousins MM, Seidler FJ (2004) Alpha7 nicotinic acetylcholine receptors targeted by cholinergic developmental neurotoxicants: nicotine and chlorpyrifos. Brain Res Bull 64(3):227–235 15464859 10.1016/j.brainresbull.2004.07.005

[46] Li Y, Xiao D, Dasgupta C et al (2012) Perinatal nicotine exposure increases vulnerability of hypoxic–ischemic brain injury in neonatal rats. Stroke 43(9):2483–2490 22738920 10.1161/STROKEAHA.112.664698 PMC3429721

[47] Rishel CW, Greeno C, Marcus SC, Shear MK, Anderson C (2005) Use of the child behavior checklist as a diagnostic screening tool in community mental health. Res Social Work Pract 15(3):195–203

[48] Papachristou E, Schulz K, Newcorn J, Bédard AC, Halperin JM, Frangou S (2016) Comparative evaluation of child behavior checklist-derived scales in children clinically referred for emotional and behavioral dysregulation. Front Psychiatry 7:146 27605916 10.3389/fpsyt.2016.00146 PMC4995201

[49] Meyer SE, Carlson GA, Youngstrom E et al (2009) Long-term outcomes of youth who manifested the CBCL-pediatric bipolar disorder phenotype during childhood and/or adolescence. J Affect Disord 113(3):227–235 18632161 10.1016/j.jad.2008.05.024

[50] Naninck EFG, Stijger PC, Brouwer-Brolsma EM (2019) The importance of maternal folate status for brain development and function of offspring. Adv Nutr 10(3):502–519 31093652 10.1093/advances/nmy120 PMC6520042

[51] Surén P, Roth C, Bresnahan M et al (2013) Association between maternal use of folic acid supplements and risk of autism spectrum disorders in children. JAMA 309(6):570–577 23403681 10.1001/jama.2012.155925 PMC3908544

[52] Zhong C, Tessing J, Lee BK, Lyall K (2020) Maternal dietary factors and the risk of autism spectrum disorders: a systematic review of existing evidence. Autism Res 13(10):1634–1658 33015977 10.1002/aur.2402 PMC9234972

[53] Binns C, Lee M, Low WY (2016) The long-term Public Health benefits of Breastfeeding. Asia Pac J Public Health 28(1):7–14 26792873 10.1177/1010539515624964

